# Shared genetic architecture of obesity and gastroesophageal reflux disease

**DOI:** 10.1097/MD.0000000000047404

**Published:** 2026-02-06

**Authors:** Lei Xiao, Dandan Zhu, Wei Liu

**Affiliations:** a Department of Thyroid Surgery, Xiangya Hospital, Central South University, Changsha, Hunan, China; b Department of Oncology, NHC Key Laboratory of Cancer Proteomics & State Local Joint Engineering Laboratory for Anticancer Drugs, National Clinical Research Center for Geriatric Disorders, Xiangya Hospital, Central South University, Changsha, Hunan, China; c Department of Gastrointestinal Surgery, Xiangya Hospital, Central South University, Changsha, Hunan, China; d Department of National Clinical Research Center for Geriatric Disorders, National Clinical Research Center for Geriatric Disorders, Xiangya Hospital, Central South University, Changsha, Hunan, China.

**Keywords:** gastroesophageal reflux disease, genome-wide pleiotropic association, obesity

## Abstract

Obesity is identified as a risk factor of gastroesophageal reflux disease (GERD). This study aims to elucidate the shared genetic architecture of obesity-related phenotypes and GERD. Based on the publicly available genome-wide association studies’ datasets, this genome-wide pleiotropic association study was conducted with various genetic approaches (including linkage disequilibrium score regression, high-definition likelihood inference for genetic correlations, pleiotropic analysis under composite null hypothesis, Functional Mapping and Annotation, Bayesian colocalization, summary-based Mendelian randomization, and multi-marker analysis of genomic annotation analysis) sequentially to unravel the genetic associations from single-nucleotide polymorphism to gene levels, and to reveal the underlying shared genetic architecture between obesity-related phenotypes and GERD. This study discovered shared genetic mechanisms between GERD and several obesity-related phenotypes, including arm fat percentage (left), arm fat percentage (right), leg fat percentage (left), leg fat percentage (right), trunk fat percentage, waist-to-hip ratio, and body mass index. Significant genetic correlations were observed by linkage disequilibrium score regression and high-definition likelihood inference for genetic correlations, with multiple associated pleiotropic loci and their mapped genes identified by pleiotropic analysis under composite null hypothesis, Functional Mapping and Annotation, Bayesian colocalization, summary-based Mendelian randomization, and multi-marker analysis of genomic annotation analysis. Additionally, several brain tissues were identified to be linked to both obesity and GERD by multi-marker analysis of genomic annotation. This research provided strong evidence of genetic correlations and brought novel insights into the underlying genetic connections and shared genetic architectures of obesity and GERD.

## 1. Introduction

Gastroesophageal reflux disease (GERD), following the Montreal classification criteria, is characterized as a condition that reflux of stomach contents induces bothersome, annoying symptoms and/or complications.^[1]^ Heartburn, chest discomfort, and regurgitation are common symptoms of GERD. Additionally, a broad range of manifestations, such as persistent coughing, wheezing, dental erosions, globus sensation, and posterior laryngitis, have been demonstrated to be associated with GERD.^[2]^ Transient lower esophageal sphincter relaxations, hiatal hernias, abnormalities in sphincter pressure, visceral hypersensitivity, and delayed gastric emptying were proposed pathogenesis of GERD.^[3]^ About 13.3% of people worldwide suffer from GERD, and the annual cost of GERD in the USA is estimated at $10 billion.^[4,5]^ Obesity was widely recognized as an independent risk factor of GERD;^[4–6]^ however, because of various constraints, the genetic mechanisms by which obesity influences the formation of GERD have not been investigated yet.

Genome-wide association studies (GWAS) can investigate genetic associations of different genotypes and phenotypes by analyzing variations in allele frequencies among ancestrally similar individuals who display distinct phenotypic traits.^[7]^ Although GWAS have successfully identified numerous genetic loci associated with obesity and GERD individually,^[8–10]^ the shared genetic architecture underlying their well-established epidemiological relationship remains largely unexplored. While observational studies consistently link obesity to an increased risk of GERD, the specific genetic mechanisms mediating this connection are not yet understood. Advances in GWAS and genetic methodologies have now made it possible to address the knowledge gap regarding pleiotropic genetic determinants and the biological pathways through which obesity influences GERD pathogenesis. Accordingly, we conducted a genome-wide cross-trait analysis using a series of statistical genetic techniques described above sequentially to uncover the shared genetic etiology between several obesity-related traits and GERD.

## 2. Methods

### 2.1. GWAS summary data acquisition and quality control

All of the GWAS data of obesity-related phenotypes and GERD were sourced from publicly accessible datasets with individuals of the European ancestry. We employed 9 obesity-related phenotypes, including arm fat percentage (left) (AFPL), arm fat percentage (right) (AFPR), leg fat percentage (left) (LFPL), leg fat percentage (right) (LFPR), trunk fat percentage (TFP), hip circumference (HC), waist circumference (WC), waist-to-hip ratio (WHR), and body mass index (BMI). The detailed information of GWAS summary data used above were presented in Table 1.

**Table 1:** The characteristics of GWAS datasets.

Phenotypes	Sample size (case/control)	Ancestry	Date source
GERD	32,232/385,082	European	FinnGen biobank
AFPL	413,046	European	UK Biobank
AFPR	413,107	European	UK Biobank
LFPL	413,138	European	UK Biobank
LFPR	413,161	European	UK Biobank
TFP	412,944	European	UK Biobank
HC	225,487	European	25673412*
WC	245,746	European	25673412*
WHR	224,459	European	25673412*
BMI	681,275	European	30124842*

AFPL = arm fat percentage (left), AFPR = arm fat percentage (right), BMI = body mass index, GERD = gastroesophageal reflux disease, GWAS = genome-wide association studies, HC = hip circumference, LFPL = leg fat percentage (left), LFPR = leg fat percentage (right), TFP = trunk fat percentage, WC = waist circumference, WHR = waist-to-hip ratio.

* The PMID number of the data source.

To assure the accuracy and reliability of these GWAS summary statistics used in our study, single-nucleotide polymorphisms (SNPs) in the major histocompatibility complex region, specifically the 25 to 35 Mb interval on chromosome 6, were excluded. This region, known for its complex gene structure and extensive linkage disequilibrium, could result in false-positive results potentially.

All genetic data used in this study were obtained from publicly available GWAS databases. No direct participant consent was obtained by the authors, as all analyses were conducted using summary-level data from previously published studies that had obtained appropriate ethical approvals and participant consent.

### 2.2. Genetic correlation analysis

Linkage disequilibrium score regression (LDSC)^[11]^ and the high-definition likelihood inference for genetic correlations (HDL)^[12]^ have been developed to analyze genetic correlations among complex traits. In our study, these methods were employed to explore genome-wide genetic correlations between obesity-related phenotypes described above and GERD. HDL analysis has been shown to reduce the variance in genetic correlation estimates by approximately 60% compared to LDSC. A *P* value <0.05 denotes statistical significance, and *r*_g_ represents the genetic correlation between trait pairs. To further enhance the reliability and accuracy of our results, Bonferroni correction was performed in this study.

### 2.3. Identification of pleiotropic loci and genes of obesity-related phenotypes and GERD – pleiotropic analysis under composite null hypothesis, Functional Mapping and Annotation, Bayesian colocalization, and multi-marker analysis of genomic annotation analysis

The novel pleiotropic analysis under composite null hypothesis (PLACO) method,^[13]^ by which using a level-α intersection-union test was developed recently to detect pleiotropy between different traits at the SNP level, was employed to explore potential pleiotropy SNPs of obesity-related phenotypes and GERD. Significant pleiotropic variants were identified as SNPs recognized by PLACO with a *P* value reach the genome-wide significant threshold (*P* < 5E−08). And then we utilized the Functional Mapping and Annotation (FUMA) tool^[14]^ to identify the genomic regions associated with these risk variants. A Bayesian colocalization analysis^[15]^ was further conducted to determine pleiotropic loci shared by obesity-related phenotypes and GERD. To further investigate the shared mechanisms of these identified pleiotropic loci, nearest genes were mapped based on the lead SNPs identified by FUMA tool within each locus. The multi-marker analysis of genomic annotation (MAGMA) method^[16]^ was employed to determine the biological function of these pleiotropic loci. Specifically, we utilized the MAGMA gene analysis to explore potential pleiotropic genes properly by incorporating linkage disequilibrium between the markers and detecting multi-marker effects. Additional gene-set analysis was employed to explore the biological functions of the identified lead SNPs; a total number of 10,678 gene sets, including curated (c2.all) and Gene Ontology terms (c5.bp, c5.cc, c5.mf), from the Molecular Signatures Database were tested ultimately.^[17]^ To further reduce false-positive rate, Bonferroni correction was conducted in the MAGMA gene and gene-set test. Finally, a genome-wide tissue-specific enrichment analysis using 54 Genotype-Tissue Expression tissues based on the genome-wide pleiotropic results identified by PLACO was performed.^[18]^ The average expression (log2 transformed) of all identified pleiotropic genes across the 54 tissues was calculated, and tissue specificity was assessed through differentially expressed genes. Differentially expressed genes (both upregulated and downregulated differentially expressed genes) were precomputed using the signs of *t*-statistics for each tissue.

### 2.4. Summary‑based Mendelian randomization

The summary-based Mendelian Randomization (SMR)^[19]^ method integrates summary-level data from GWAS with expression quantitative trait loci (eQTL) studies to detect genes whose expression levels are associated with complex traits through pleiotropy. We employed SMR, combined with Heterogeneity Estimation Detects Independent Instruments methods, to evaluate pleiotropic associations between gene expression and complex traits using summary-level GWAS and eQTL data. This analysis could test whether SNP effects on a phenotype are mediated through gene expression.

### 2.5. Software and packages

In our study, the primary statistical analysis was performed in R (version 4.2.2; The R Foundation for Statistical Computing, Vienna, Austria, https://www.r-project.org/). LDSC analysis utilized the “LDSC” software (v1.0.1; Developed by the Bulik–Sullivan Lab [Broad Institute of MIT and Harvard], Cambridge, https://github.com/bulik/ldsc),^[11]^ while PLACO analysis for obesity-related phenotypes and GERD was performed using the “PLACO” package.^[13]^ Bayesian colocalization analysis employed the “coloc” package^[15]^ (v5.2.1; Developed by Dr Chris Wallace and colleagues [University of Cambridge], Cambridge, United Kingdom, https://cran.r-project.org/package=coloc). Functional annotation and MAGMA analysis were carried out via the FUMA web tool and MAGMA software.^[14,16]^ Additionally, SMR analysis was executed using the “SMR” software.^[19]^

## 3. Results

### 3.1. Genetic correlation between obesity-related phenotypes and GERD – LDSC and HDL

The LDSC and HDL methods were employed to assess the genetic correlation between 9 obesity-related phenotypes and GERD, the results from these 2 methods were highly consistent, highlighting the reliability of our findings. AFPL, AFPR, LFPL, LFPR, TFP, WHR, and BMI were identified to be positively genetical correlated with GERD, while HC was detected to be negatively genetical correlated with GERD. No significant genetic correlation between WC and GERD was identified by LDSC and HDL analysis in this study. It was worth noting that AFPL, AFPR, LFPL, LFPR, TFR, WHR, and BMI remained genetically correlated with GERD even if the Bonferroni correction was applied in both of the LDSC and HDL results. The results of genetic correlation analysis using LDSC and HDL methods were detailed in Tables 2 and S1 and S2, Supplemental Digital Content, https://links.lww.com/MD/R289. Considering that no positively genetic correlation was found between HC and GERD (as well as WC and GERD), these 2 trait pairs were excluded in our further analysis.

**Table 2:** Genetic correlation between obesity-related phenotypes and GERD.

Trait pairs	LDSC			HDL		
	*r*_g_ (SE)	*P*	*P*_bon	*r*_g_ (SE)	*P*	*P*_bon
GERD–AFPL	0.152 (0.025)	9.82E−10	8.84E−09	0.188 (0.027)	2.13E−12	1.92E−11
GERD–AFPR	0.152 (0.025)	2.01E−09	1.81E−08	0.194 (0.026)	6.66E−14	5.99E−13
GERD–LFPL	0.213 (0.026)	1.72E−16	1.55E−15	0.257 (0.030)	2.23E−17	2.01E−16
GERD–LFPR	0.217 (0.026)	2.60E−17	2.34E−16	0.260 (0.030)	1.13E−17	1.02E−16
GERD–TFP	0.143 (0.025)	8.39E−09	7.55E−08	0.189 (0.028)	1.67E−11	1.50E−10
GERD–HC	−0.104 (0.033)	1.51E−03	0.014	−0.148 (0.042)	3.66E−04	3.29E−03
GERD–WC	0.036 (0.041)	0.375	1	0.059 (0.047)	0.211	1
GERD–WHR	0.169 (0.043)	7.54E−05	6.78E−04	0.241 (0.061)	7.21E−05	6.49E−04
GERD–BMI	0.118 (0.025)	2.92E−06	2.62E−05	0.123 (0.026)	1.35E−06	1.22E−05

AFPL = arm fat percentage (left), AFPR = arm fat percentage (right), BMI = body mass index, GERD = gastroesophageal reflux disease, HC = hip circumference, HDL = high-definition likelihood inference for genetic correlations, LDSC = linkage disequilibrium score regression, LFPL = leg fat percentage (left), LFPR = leg fat percentage (right), *P*_bon = *P* value after Bonferroni correction, TFP = trunk fat percentage, WC = waist circumference, WHR = waist-to-hip ratio.

### 3.2. *Identification of pleiotropic, genomic risk loci of GERD and obesity-related phenotypes* – *PLACO, FUMA, and Bayesian colocalization analysis*

Given the shared genetic mechanisms identified by LDSC and HDL between obesity-related phenotypes (AFRL, AFRR, LFRL, LFRR, TFR, BMI, and WHR) and GERD, the novel pleiotropy analyses (PLACO) was employed to find potential pleiotropic loci for obesity-related phenotypes and GERD (p.placo < 5E–8 and T.paco > 0). A total of 43 SNPs were identified as potential pleiotropic loci between BMI and GERD (Table S3, Supplemental Digital Content, https://links.lww.com/MD/R289), while 48 pleiotropic SNPs were identified between WHR and GERD (Table S4, Supplemental Digital Content, https://links.lww.com/MD/R289). A total of 4074 pleiotropic SNPs were identified between GERD and 5 body-fat percentage phenotypes (AFRL, AFRR, LFRL, LFRR, and TFR); notably, 88 repeated pleiotropic SNPs were identified across these 5 trait pairs (Tables S5–S10, Supplemental Digital Content, https://links.lww.com/MD/R289), indicating a comprehensive shared genetic architecture between GERD and body-fat percentage. Based on the PLACO results, FUMA tool identified a total of 137 independent genomic risk loci (*P* < 5E–8) associated with GERD and obesity-related phenotypes (Table S11, Supplemental Digital Content, https://links.lww.com/MD/R289). Additional Bayesian colocalization analysis finally identified 33 of the 137 (24.1%) potential colocalized pleiotropic loci with posterior probability of H4 >0.7 (Tables 3 and S12, Supplemental Digital Content, https://links.lww.com/MD/R289).

**Table 3:** Thirty-three colocalized loci in Bayesian colocalization analysis based on 137 genomic risk loci identified by FUMA tool (PP.H4 > 0.7).

Trait pairs	Locus boundary	Region	Lead SNPs	*P*	PP.H4
GERD–AFPL	7:26,835,508–27,131,573	7p15.2	rs213519	2.86E−08	0.857
GERD–AFPL	8:143,752,994–143,780,261	8q24.3	rs2717609	9.02E−10	0.941
GERD–AFPL	11:27,719,422–27,719,422	11p14.1	rs76324918	1.40E−09	0.765
GERD–AFPL	18:21,074,255–21,165,409	18q11.2	rs1788808	6.18E−12	0.808
GERD–AFPL	19:18,806,668–18,834,514	19p13.11	rs8112818	1.87E−10	0.702
GERD–AFPR	6:50,427,682–50,985,925	6p12.3	rs72887104; rs546979	2.62E−11	0.700
GERD–AFPR	7:26,835,508–27,131,573	7p15.2	rs774266	1.62E−08	0.855
GERD–AFPR	8:143,752,994–143,780,261	8q24.3	rs2717609	1.37E−09	0.942
GERD–AFPR	11:27,719,422–27,719,422	11p14.1	rs76324918	2.13E−09	0.765
GERD–AFPR	18:21,074,255–21,165,409	18q11.2	rs1788808	6.59E−12	0.828
GERD–AFPR	19:18,806,668–18,834,514	19p13.11	rs8112818	8.94E−11	0.761
GERD–LFPL	3:50,181,135–50,181,135	3p21.31	rs2526385	3.00E−08	0.915
GERD–LFPL	8:143,752,994–143,825,951	8q24.3	rs1045547	2.82E−08	0.912
GERD–LFPL	11:27,719,422–27,719,422	11p14.1	rs76324918	9.95E−10	0.765
GERD–LFPL	18:21,074,255–21,165,409	18q11.2	rs1788781	1.28E−11	0.863
GERD–LFPL	20:44,875,993–44,907,944	20q13.12	rs2425805	3.85E−08	0.943
GERD–LFPR	3:50,181,135–50,181,135	3p21.31	rs2526385	3.27E−08	0.914
GERD–LFPR	5:42,896,946–42,915,470	5p12	rs17240372	3.21E−08	0.787
GERD–LFPR	6:50,664,180–50,985,925	6p12.3	rs74388152; rs546979	2.86E−11	0.730
GERD–LFPR	8:143,752,994–143,780,261	8q24.3	rs2717609	8.11E−09	0.934
GERD–LFPR	11:27,719,422–27,719,422	11p14.1	rs76324918	6.41E−10	0.765
GERD–LFPR	18:21074,255–21165409	18q11.2	rs1788781	1.50E−11	0.876
GERD–TFP	3:50,181,135–50,181,135	3p21.31	rs2526385	2.01E−08	0.916
GERD–TFP	6:50,620,566–50,985,925	6p12.3	rs74388152; rs546979	8.64E−11	0.749
GERD–TFP	7:26,835,508–27,131,573	7p15.2	rs774266	2.27E−09	0.870
GERD–TFP	8:143,752,994–143,780,261	8q24.3	rs2717609	3.83E−10	0.939
GERD–TFP	18:21,074,255–21,165,409	18q11.2	rs1788781	2.22E−11	0.712
GERD–TFP	19:18,806,668–18,834,514	19p13.11	rs8112818	1.18E−09	0.752
GERD–WHR	17:61,646,000–62,013,474	17q23.3	rs12325866	6.69E−09	0.731
GERD–BMI	2:57,943,567–58,093,903	2p16.1	rs1106090	2.73E−08	0.937
GERD–BMI	5:43,054,747–43,193,052	5p12	rs10075647	3.86E−08	0.702
GERD–BMI	17:27,935,546–28,550,732	17q11.2	rs1038088	3.13E−08	0.893
GERD–BMI	18:21,074,922–21,165,163	18q11.2	rs1788823	4.94E−09	0.815

AFPL = arm fat percentage (left), AFPR = arm fat percentage (right), BMI = body mass index, FUMA = Functional Mapping and Annotation, LFPL = leg fat percentage (left), LFPR = leg fat percentage (right), PP.H4 = posterior probability of H4, SNP = single-nucleotide polymorphism, TFP = trunk fat percentage, WHR = waist-to-hip ratio.

### 3.3. Identification of pleiotropic gene, gene-set and additional tissue-specific analysis – SMR and MAGMA

**Figure 1.**
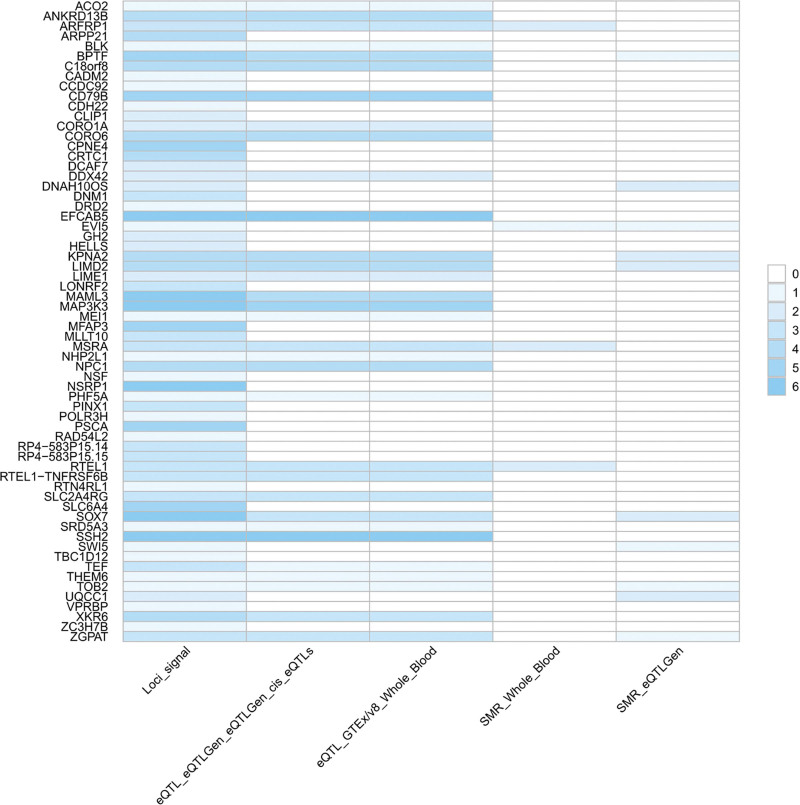
Overview of pleiotropic genes identified by the convergence of PLACO, eQTL, and SMR analyses. The genes listed here were identified through an integrative analysis of the shared genetics between obesity-related phenotypes and GERD. eQTL = expression quantitative trait loci, GERD = gastroesophageal reflux disease, PLACO = pleiotropic analysis under composite null hypothesis, SMR = summary-based Mendelian randomization.

**Figure 2.**
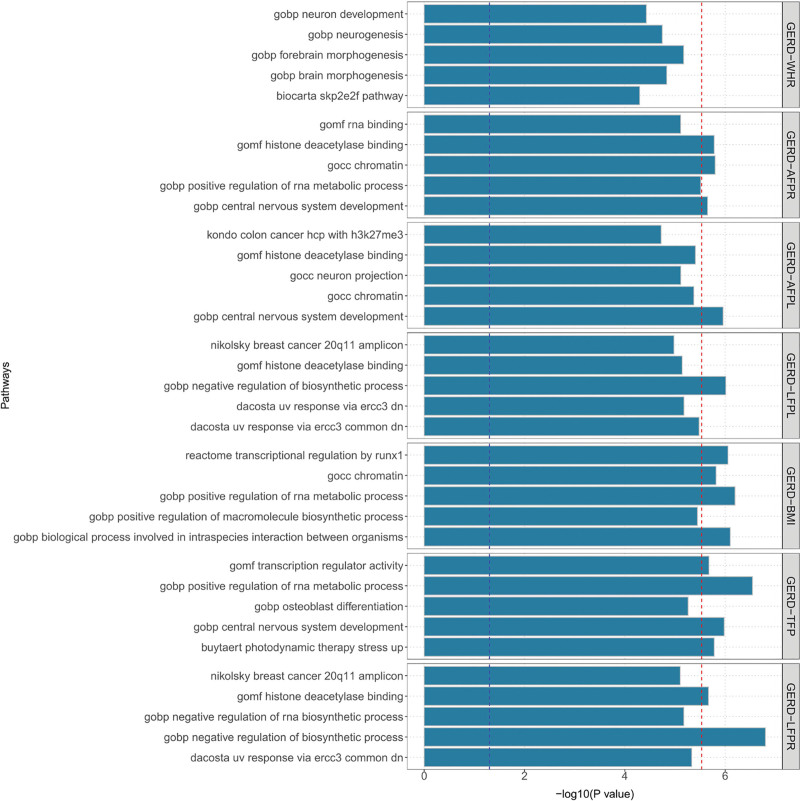
MAGMA gene-set analysis for genome-wide pleiotropic obesity-GERD gene. Note: The plot visualizes the enrichment of genome-wide pleiotropic signals in curated biological pathways. Significance thresholds are indicated by the blue line (nominal *P* < .05) and the red line (Bonferroni-corrected *P* < .05). Pathways exceeding the red threshold are significantly enriched and pinpoint key biological mechanisms shared between obesity and GERD. GERD = gastroesophageal reflux disease, MAGMA = multi-marker analysis of genomic annotation.

**Figure 3.**
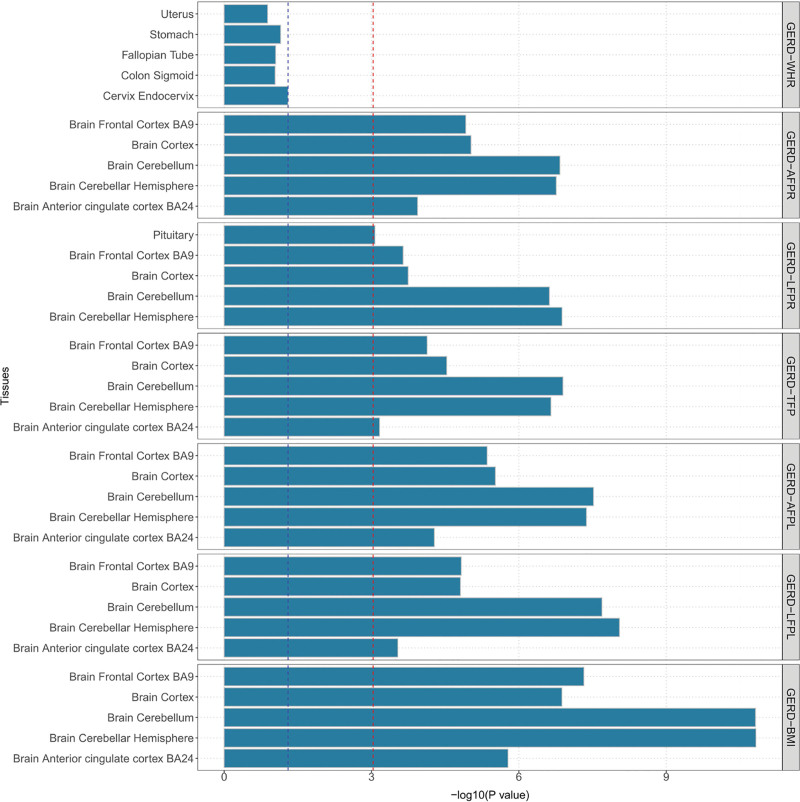
MAGMA tissue-specific analysis for pleiotropic obesity–GERD genes identified by MAGMA analysis. Note: This plot presents the results of the MAGMA tissue-specific expression analysis, which tests whether genes from pleiotropic loci between obesity-related phenotypes and GERD are highly expressed in specific human tissues. The blue horizontal line indicates the nominal significance threshold (*P* = .05), and the red horizontal line marks the study-wide significance threshold after Bonferroni correction for multiple testing across all examined tissues. GERD = gastroesophageal reflux disease, MAGMA = multi-marker analysis of genomic annotation.

We employed several methods to map the identified SNP-level pleiotropic loci into the gene-level signals. A total of 180 significant (after Bonferroni correction) genes were identified to be pleiotropic genes between obesity-related phenotypes and GERD (Table S13, Supplemental Digital Content, https://links.lww.com/MD/R289 and Figs. S1–S7, Supplement Digital Content, https://links.lww.com/MD/R290) by MAGMA. Specifically, *CPNE4*, EF-Hand Calcium Binding Domain 5 (*EFCAB5*), *MAML3*, Nuclear Speckle Splicing Regulatory Protein 1 (*NSRP1*), *PSCA*, *SLC6A4*, and Slingshot Protein Phosphatase 2 (*SSH2*) were repeated pleiotropic genes between GERD and 5 body-fat percentage phenotypes. In these 7 repeated pleiotropic genes, *MAML3*, *NSRP1*, *EFCAB5*, and *SSH2* were also pleiotropic genes of BMI and GERD. Additionally, *MAP3K3* was identified as a potential pleiotropic gene for 6 trait pairs, followed by *CD79B*, *MFAP3*, and *SOX7* as pleiotropic genes in 5 trait pairs. We summarized the landscape of these pleiotropic genes identified in different methods (PLACO, eQTL, and SMR) and tissues in Figure 1. It is worth noting that 2 genes (*EFCAB5* and *SSH2*) were mapped significantly in different tissues with these different methods. The MAGMA gene-set analysis suggested that the identified lead SNPs may be involved in central nervous system development, positive regulation of RNA metabolic process, etc (after Bonferroni correction, Fig. 2 and Table S14, Supplemental Digital Content, https://links.lww.com/MD/R289). Additional tissue-specific analysis by MAGMA found these lead SNPs were enriched in several structures of the brain, such as frontal cortex, cortex, cerebellum, cerebellar hemisphere, and anterior cingulate cortex (after Bonferroni correction, Fig. 3 and Table S15, Supplemental Digital Content, https://links.lww.com/MD/R289). However, no specific tissues were enriched between GERD and WHR after applying the Bonferroni correction.

## 4. Discussion

To the best of our knowledge, our study represents the first large-scale genome-wide cross-trait analysis that employs various statistical genetic techniques sequentially to uncover the shared genetic architecture underlying GERD and obesity. Positive overall genetic correlations were identified by LDSC and HDL analysis between GERD and AFPL, AFPR, LFPL, LFPR, TFP, BMI, as well as WHR. A number of potential pleiotropic loci were detected through PLACO, FUMA, and additional Bayesian colocalization analysis. We mapped the identified SNP-level pleiotropic loci to the gene-level signals between GERD and obesity. A set of pleiotropic genes were identified; further functional enrichment analysis and tissue-specific analysis were also performed, providing insights into the biological processes underlying GERD and obesity.

The findings regarding genetic correlations between obesity and GERD from this study are largely in line with previous conventional epidemiological studies, yet provide some novel insights in several aspects. A meta-analysis found that obesity was significantly associated with GERD,^[4]^ through genetic correlation analysis using LDSC and HDL, we identified significant genetic overlap between GERD and 7 obesity-related phenotypes (BMI, WHR, and 5 body-fat percentage phenotypes: AFPL, AFPR, LFPL, LFPR, and TFP). Gain in BMI was identified as a risk factor of GERD in a HUNT Study,^[5]^ and higher adulthood BMI was genetically associated with an increased risk of GERD in a Mendelian randomization study.^[20]^ However, these previous studies did not have a chance for directly assessing the genetic correlation between BMI and GERD using LDSC and HDL analysis. Visceral fat is considered more harmful than fat located in other body regions.^[21]^ Limited by the time-consuming and expensive technique, visceral adiposity is often evaluated by WHR in observational studies. Corroborating previous observational studies, our study demonstrated that WHR was positively genetically correlated with GERD, unlike HC or WC. Body-fat percentage was associated with GERD in previous observational study, but no causal association was observed in previous Mendelian randomization study.^[22]^ We conducted a comprehensive analysis and identified that all the 5 body-fat percentage phenotypes (AFPL, AFPR, LFPL, LFPR, and TFP) were genetically correlated with GERD, which is consistent with the results of previous observational studies.

Based on the shared genetic mechanisms identified by LDSC and HDL, we further detected a set of pleiotropic loci and genes sequentially. Five genes (*MAML3*, *NSRP1*, *EFCAB5*, *SSH2*, and *MAP3K3*) were identified as novel pleiotropic genes between GERD and obesity (involving at least 6 trait pairs). In a previous study, *MAML3* was identified as a pleiotropic gene associated with both GERD and smoking behavior.^[23]^ To date, no associations have been established between *NSRP1*, *EFCAB5*, or *SSH2* and *GERD* or obesity, with existing literature primarily linking these genes to neurodevelopmental disorder,^[24]^ autism spectrum disorder,^[25]^ or Alzheimer’s disease.^[26]^ Notably, *EFCAB5* and *SSH2* were also identified as pleiotropic genes in the eQTL analysis of obesity and GERD, which provides new insights for developing relevant medications for the treatment or prevention of GERD.

Tissue-specific enrichment analysis revealed varying degrees of enrichment of GERD and obesity-related phenotypes across several different brain tissues, with the brain’s cortex and brain’s cerebellum showing the most significant associations. The ongoing development and understanding of the “brain–gut” axis in psychogastroenterology have substantially advanced insights into chronic digestive diseases.^[27]^ The integration of the exogenous autonomic nervous system with gut function through excitatory sympathetic and vagal pathways is essential for maintaining lower esophageal sphincter function.^[28]^ Previous studies by Shaker^[29]^ and Wang et al^[30]^ demonstrated heightened insular cortex activity during esophageal stimulation in GERD patients, indicating a sensitive esophageal–cortical axis. An increased prevalence of neural dysfunction has been reported in individuals with obesity.^[31–33]^ Thus, the substantial impact of obesity on the nervous system may represent a mechanism linking GERD and obesity through neural pathways, highlighting the relevance of the identified brain tissues.

Our study also has several certain limitations. First, like other GWAS studies, this research relied on summary-level data due to the unavailability of individual-level datasets. Second, our study did not include further population stratification by age or gender. Third, our data were limited to individuals of the European ancestry, which may limit the applicability of our findings to other ethnic groups (e.g., the African and Asian populations).

## 5. Conclusion

In summary, this research provided significant evidence of genetic correlation between obesity and GERD, with multiple associated pleiotropic loci and their mapped genes identified. Additionally, various brain tissues were identified to be linked to both obesity and GERD. These novel findings may help elucidate the underlying genetic connections and shared genetic architectures between obesity and GERD.

## Author contributions

**Conceptualization:** Dandan Zhu, Wei Liu.

**Data curation:** Lei Xiao.

**Formal analysis:** Lei Xiao.

**Funding acquisition:** Dandan Zhu, Wei Liu.

**Investigation:** Lei Xiao.

**Methodology:** Lei Xiao.

**Project administration:** Lei Xiao.

**Resources:** Lei Xiao.

**Software:** Lei Xiao.

**Supervision:** Dandan Zhu, Wei Liu.

**Validation:** Lei Xiao, Wei Liu.

**Visualization:** Lei Xiao, Wei Liu.

**Writing – original draft:** Lei Xiao, Dandan Zhu, Wei Liu.

**Writing – review & editing:** Lei Xiao, Dandan Zhu, Wei Liu.

## Supplementary Material




