# Supplementary material for: Shared genetic architecture of obesity and gastroesophageal reflux disease
Source: Medicine (Baltimore). 2026 Feb 6;105(6):e47404. doi: 10.1097/MD.0000000000047404 (PMC12885720; doi:10.1097/MD.0000000000047404)
Supplement: Supplementary file 2 [file medi-105-e47404-s002.docx]

Supplementary Figures


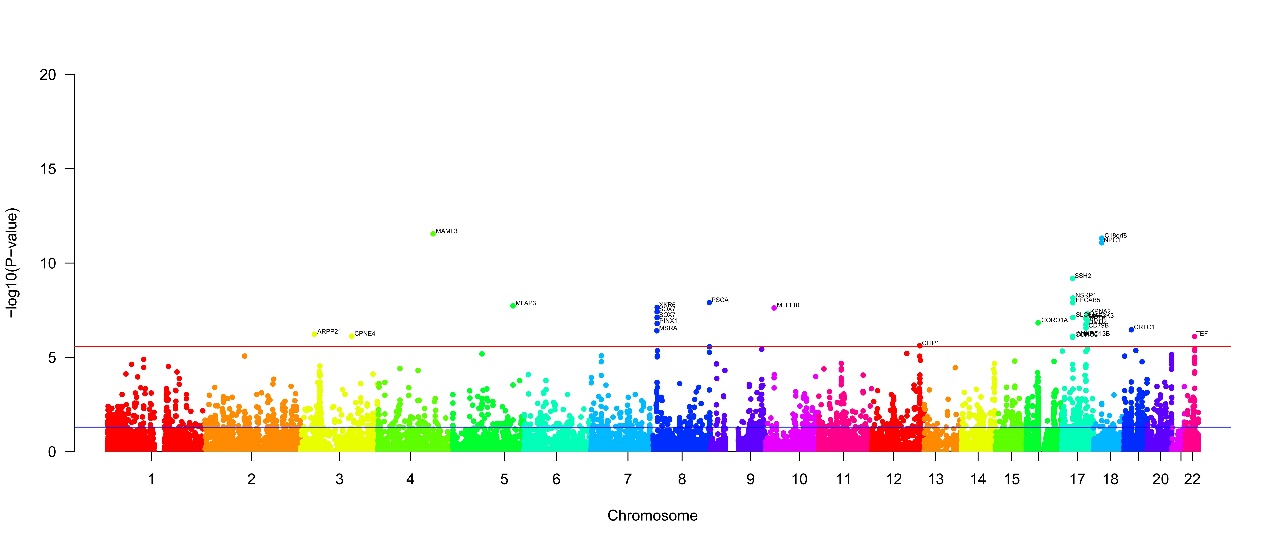


**Figure S1.** Pleiotropic genes of AFPL and GERD (geneManhattan plot). Note: Red line represents the *P*-value after Bonferroni correction.


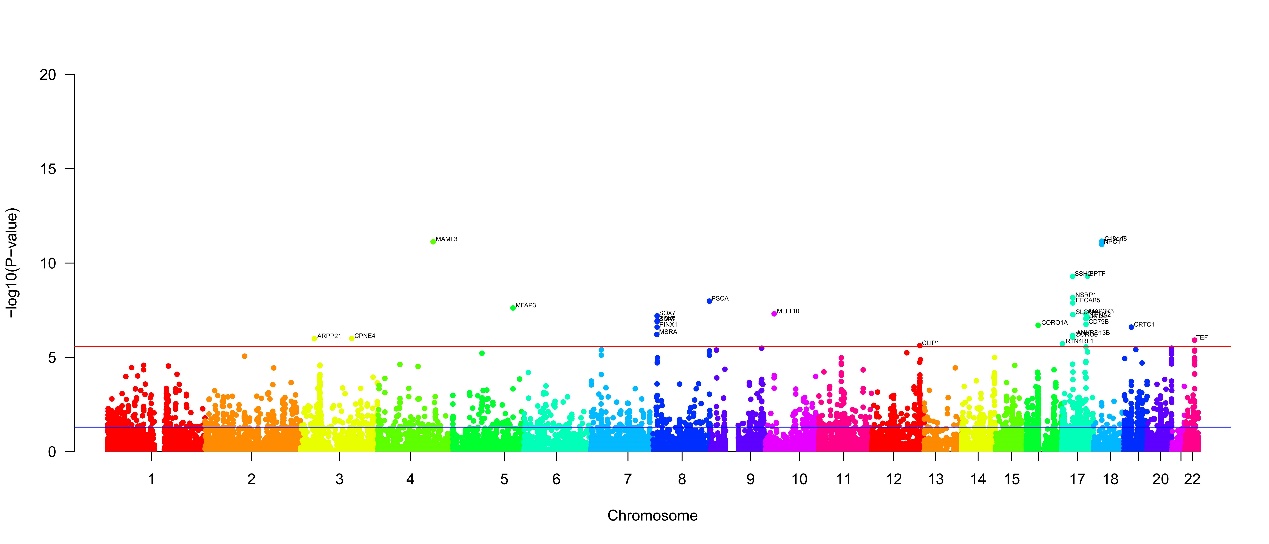


**Figure S2.** Pleiotropic genes of AFPR and GERD (geneManhattan plot). Note: Red line represents the *P*-value after Bonferroni correction.


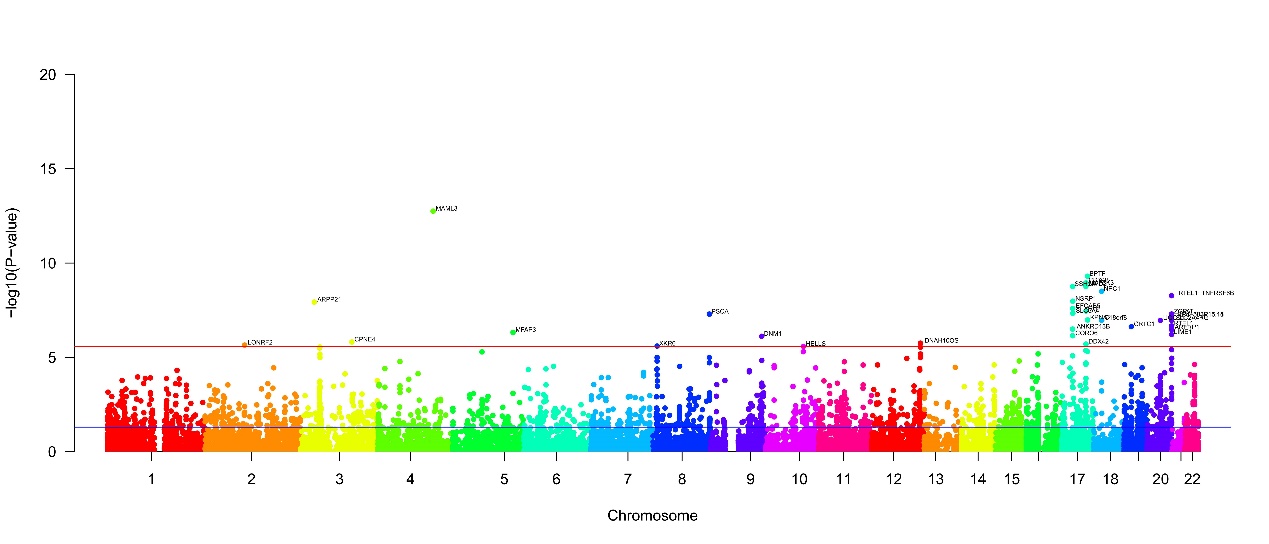


**Figure S3.** Pleiotropic genes of LFPL and GERD (geneManhattan plot). Note: Red line represents the *P*-value after Bonferroni correction.


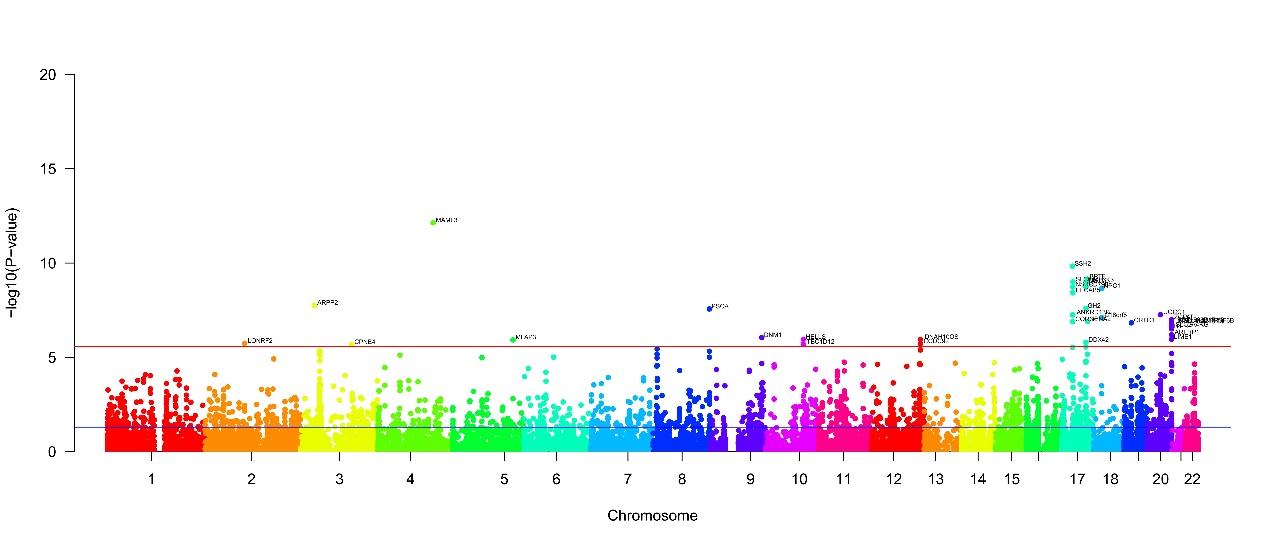


**Figure S4.** Pleiotropic genes of LFPR and GERD (geneManhattan plot). Note: Red line represents the *P*-value after Bonferroni correction.


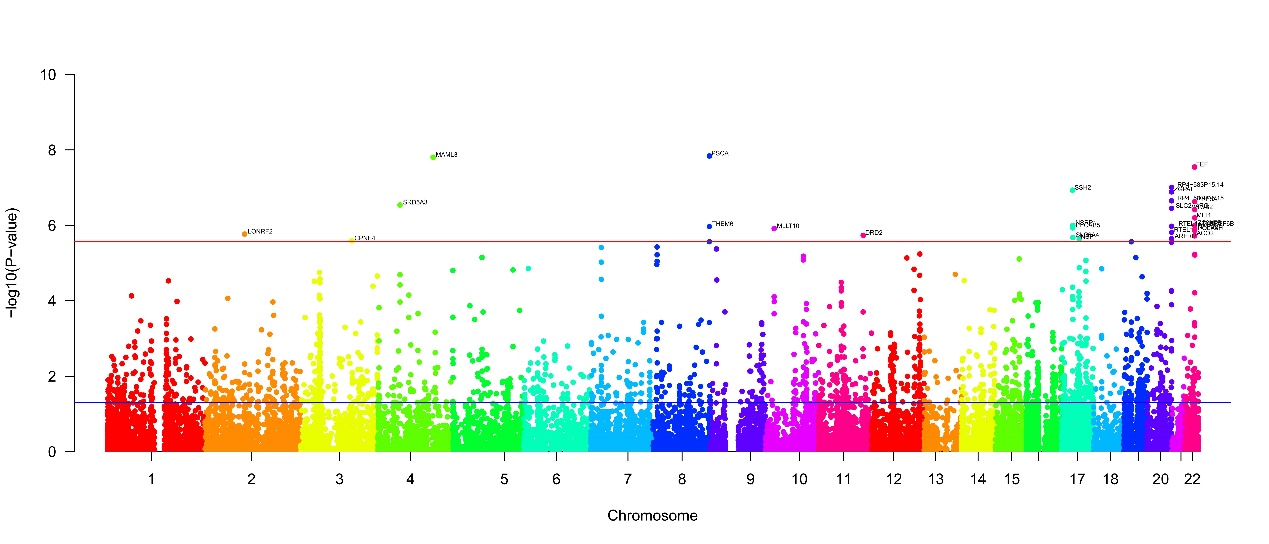


**Figure S5.** Pleiotropic genes of TFP and GERD (geneManhattan plot). Note: Red line represents the *P*-value after Bonferroni correction.


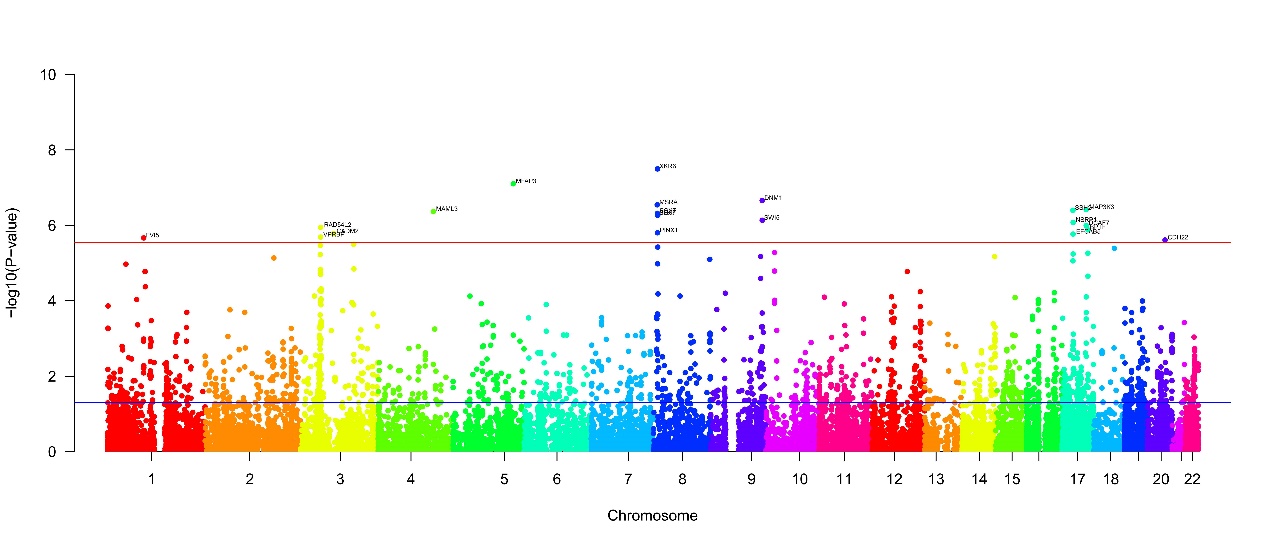


**Figure S6.** Pleiotropic genes of BMI and GERD (geneManhattan plot). Note: Red line represents the *P*-value after Bonferroni correction.


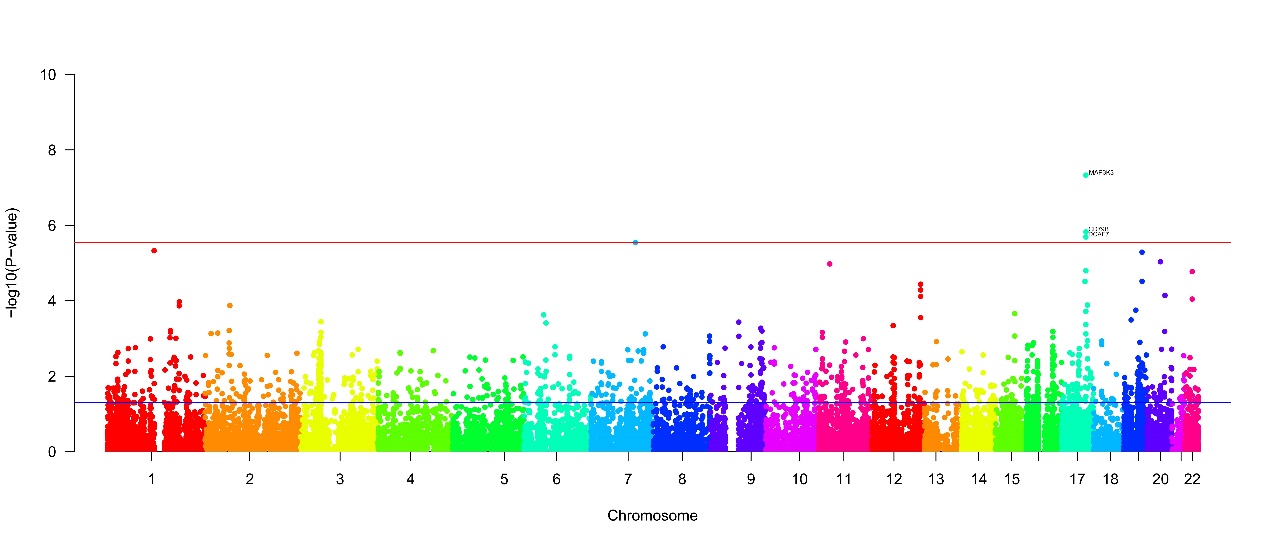


**Figure S7.** Pleiotropic genes of WHR and GERD (geneManhattan plot). Note: Red line represents the *P*-value after Bonferroni correction.
